# Fracture of the astragalus in pediatric patients, an unusual entity

**DOI:** 10.1016/j.ijscr.2021.105648

**Published:** 2021-03-05

**Authors:** Salomón Jasqui Remba, Isaac Baley Amiga, Lucero Noemi Santos Aragón, Mauricio Montalvo, Daniel Portman Santos

**Affiliations:** a Hospital Ángeles Lomas, Office 165, Mexico; b American British Cowdray Medical Center, Office 073, Mexico

**Keywords:** Astragalus fracture, Pediatric, Foot pain, Avascular necrosis, Case report

## Abstract

**Introduction and importance:**

The fracture of the astragalus is an unusual bone fracture (Ladero and Concejero, 2004) and is even more unusual in pediatric patients. The astragalus is a bone surrounded by cartilage and other structures making it difficult to appreciate on an X-ray which can lead to misdiagnosis (Ladero and Concejero, 2004; Inal and Inal, 2014) and could lead to not applying the right treatment and risk avascular necrosis or other sequelae.

**Case presentation:**

3-year-old male with foot pain and edema+++, unable to walk or stand up since a fall 2 days before, had a doubtful diagnosis after an x-ray showed no apparent bone injury. A CT scan confirmed astragulus fracture; it was then treated adequately, thus avoiding complications that could have occurred had the fracture not been diagnosed and treated correctly.

**Clinical findings and interventions and outcome:**

The limb was immobilized with a suropodalic cast for 5 weeks. No surgery was performed because of the patient's age and because the fracture was not displaced (Hawkins type 1) (Jasqui-Remba and Rodriguez-Corlay, 2016; Urrutia et al., 1999).

**Relevance and impact:**

There is a risk of misdiagnosis in these cases since the astragulus fracture might not show up in an X-ray, as in the case presented. A CT scan can detect the fracture and thus help consolidate the bone correctly. The correct differential diagnosis also reduces the risk of avascular necrosis, which increases due to poor blood supply to the astragalus.

## Highlights

• The poor blood supply of the astragalus increases the risk of avascular necrosis.
• The astragalus is composed mainly of cartilage, which reduces the risk of fracture.
• It is important to keep this fracture in mind in injuries that could compromise the astragalus.
• The physician needs to know the differential diagnoses to get the right diagnosis nd to administer the correct treatment.

## 1 Introduction

The fracture of the astragalus is an unusual bone fracture, corresponding to 0.1 to 0.85% of all fractures [1], and is even more unusual in pediatric patients, with an incidence of 0.08%. The astragalus is a hidden bone conformed by 60–70% of cartilage and is surrounded by different structures, which makes it difficult to detect on an X-ray when a fracture occurs leading to misdiagnosis [1,2] and thus to the wrong treatment.

The elaboration of this case report has been reported in line with the SCARE 2020 guidelines, making sure it is compliant with all of the criteria of the SCARE 2020 Checklists [3].

## 2 Case presentation

**Fig. 1:**
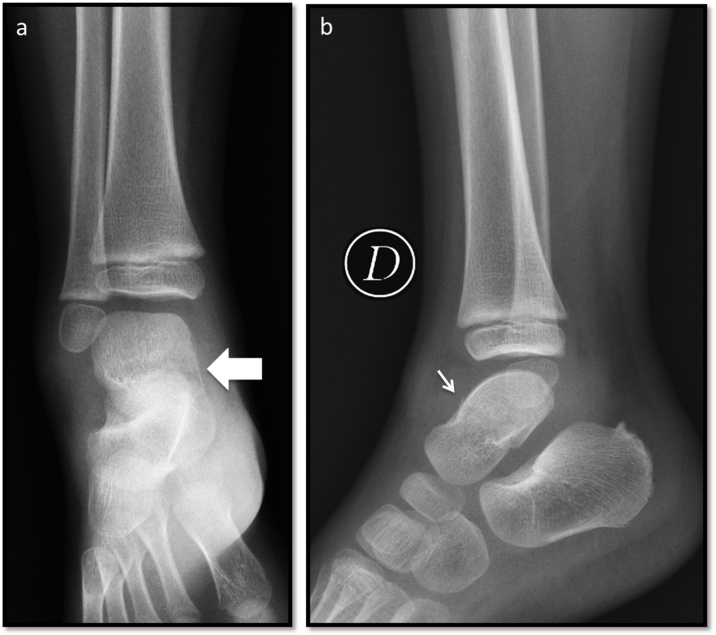
a. Anteroposterior x-ray of the ankle shows a lineal, horizontal, radiolucent image of the talus body (arrow). b. Lateral x-ray of the ankle, the radiolucent trace is less evident, shows an irregularity in the cortical bone (thin arrow).

**Fig. 2:**
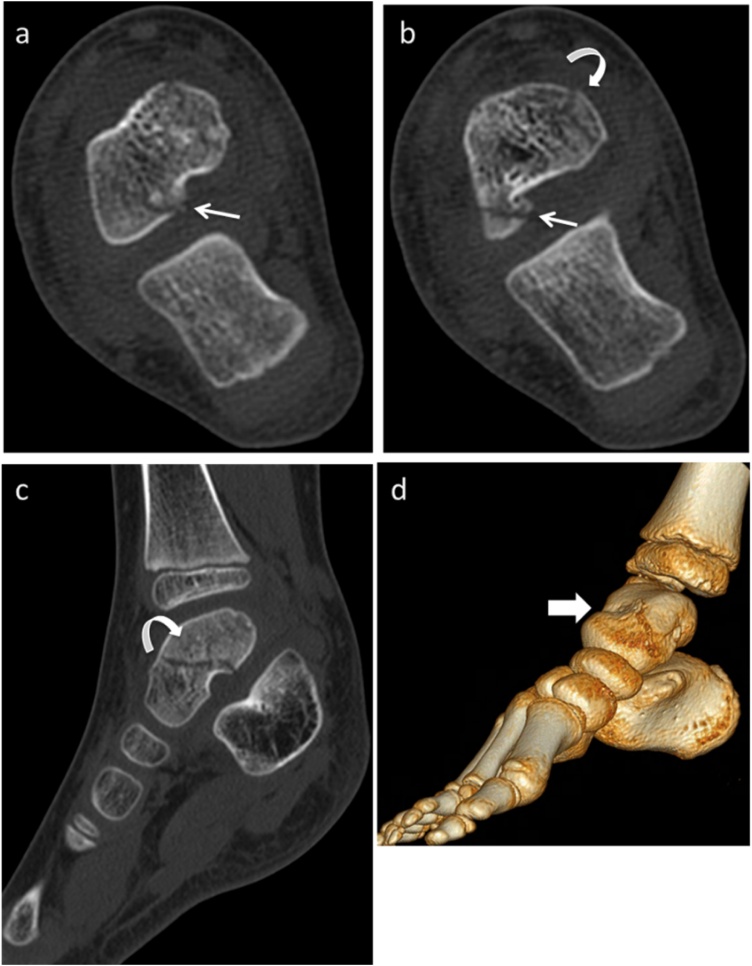
a and b. Axial CT scan shows multifragmentary fracture of the body of the astragalus (curved arrow) with intraarticular component (thin arrow). c. Sagittal reconstruction with hypodense traces of the talus body (curved arrow). d. Volumetric reconstruction shows the fracture of the talus body (arrow).

The case presented is of a 3-year-old male who attends the emergency room unable to walk and complaining of foot pain and edema +++/+++++. He reports having fallen from the third step of a staircase two days ago. Other past medical history is not relevant for the case presented. The orthopedic surgeon decided to order an x-ray which didn’t show any apparent bone injury and the diagnosis was doubtful. Fig. 1. Because of the clinical manifestations, it was necessary to perform more studies; comparative X-rays were unremarkable, so a CT scan was performed to corroborate the diagnosis. Fig. 2

### 2.1 Investigation

The incidence of fracture of the talus corresponds to 3.4% of all fractures of the foot, with 50% of them corresponding to the neck of the talus, 40% to the body, and 10% to the head; falls and traffic accidents are the most frequent causes [1,4]. It is important to take into account the poor blood supply of this bone and the way it is distributed, mainly by the posterior tibial artery and the dorsalis pedal artery, presenting a high risk of avascular necrosis, associated with 50% of the cases [5,6].

Astragalus is a bone that is 60–70% covered with articular cartilage [1]. Depending on the location of the fractures, the risk of avascular necrosis is reflected in the Hawkins scale: [5].

• Type I:
  o Vertical fractures, minimum or not displace
  o <10% risk of avascular necrosis.
• Type II:
  o Vertical and displaced fractures, with subluxated or dislocated subtalar joint
  o 42% risk of avascular necrosis.
• Type III:
  o Fracture with subtalar joint and tibiotalar dislocation
  o >90% risk of avascular necrosis.
• Type IV:
  o Type III plus talonavicular dislocation
  o >90% risk of avascular necrosis.

### 2.2 Differential diagnosis

Among the differential diagnoses, the most important ones are the triplane and Tillaux fractures. Because of the anatomical region, age of the patient, and characteristics of the injury, it can be difficult to diagnose in a case like this one and, since they can easily be confused with each other, it is always necessary to take a CT scan.

An important characteristic to consider in pediatric patients is the growth cartilage; the injuries in these structures could compromise vascularization and lead to alterations in bone growth [[7], [8], [9]].

Considering that the most common injury treated by orthopedists is the ankle fracture, and because of the closeness of the anatomical location, this pathology should be ruled out [10].

It is important to keep in mind that lesions of adjacent structures such as tendons, ligaments, vascular and nervous structures can also arise [11].

### 2.3 Treatment

The limb was immobilized by the orthopedic surgeon with a suropodalic cast for 5 weeks. No surgery was performed because of the patient's age and because the fracture was not displaced (Hawkins type 1) [12,13].

## 3 Results

### 3.1 Outcome and follow-up

After 5 weeks of treatment, the patient was able to walk without pain, and the fracture was consolidated; there was no need for physical therapy because of the patient’s age and adequate evolution.

**Fig. 3:**
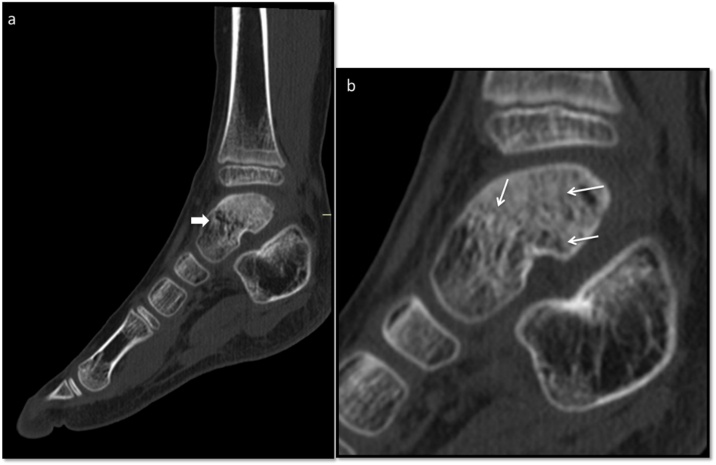
a and b. Six months after. CT scan, sagittal reconstruction with heterogenous traces of the talus body (arrow). Is evident the process of consolidation of the fracture (thin arrows).

A follow-up CT scan was done after 6 months to radiologically determine the patient’s recovery. Fig. 3.

## 4 Discussion

The astragalus fracture is an unusual entity and is even more uncommon in pediatric patients [14,15]. It should be treated timely and adequately to prevent possible complications. However, in many cases, it is not possible to have the diagnosis timely, as in the case presented by Hernández, et al. [16] of a patient who was misdiagnosed, was correctly diagnosed after one month and, by then, surgical treatment was needed. In the case presented in this article, due to the type of fracture and prompt treatment, there were no complications such as avascular necrosis or other sequelae. As Aurélien Michel-Traverso, et al. suggests, the physician should obtain all imaging studies necessary before arriving at a diagnosis [17]. In the literature there are several injury mechanisms described for a talus or astragalus fracture; a frequent one is reported in the article of Kamphuis SJ, et al. [18], where the cases presented are of different patients with dorsiflexion of the foot causing talus fracture. Seeing the same type of injury mechanism in several patients, it is possible to attribute it to the causes of this diagnosis.

According to other cases reported in the literature, it is possible to acknowledge how uncommon the astragalus fracture is, being that in many cases it is an injury often forgotten after a trauma; additionally, it can be accompanied by fractures of adjacent structures and rarely presents a sole entity.

## 5 Conclusions

The astralagus bone is composed mainly of cartilage, which reduces the risk of fracture and makes it very rare in children. However, in the case of a fracture, the risk of avascular necrosis increases because of the poor blood supply to the astragalus. Also, the astragalus can be difficult to detect on an X-ray so a CT scan may be needed to discard the possibility of fracture. It is important to keep this in mind in injuries that could compromise the astragalus so as to avoid misdiagnosis, consider the differential diagnoses, and apply the right treatment.

## Declaration of Competing Interest

All authors declare they have no conflicts of interest.

## Funding

This work does not have any type of funding or sponsors.

## Ethical approval

The study is exempt from ethical approval.

## Consent

Written informed consent was obtained from the patients parents for publication of this case report and accompanying images. A copy of the written consent is available for review by the Editor-in-Chief of this journal on request.

## Author contribution

This manuscript has been read and approved by all the authors. SJR, IBA, LNSA, MM and DPS contributed in the acquisition of the clinic patient data for the case presented, as well as the research of the related literature and the preparation of the manuscripts, LSA, IBA and DPS made the design of the study images and finally SJR, IBA and DPS made the critical revision of the manuscript.

## Registration of research studies

Not Applicable.

## Guarantor

Salomón Jasqui Remba.

## Provenance and peer review

Not commissioned, externally peer-reviewed.
